# Randomised controlled trials of antihypertensive therapy: does exclusion of orthostatic hypotension alter treatment effect? A systematic review and meta-analysis

**DOI:** 10.1093/ageing/afad044

**Published:** 2023-04-01

**Authors:** Catriona Reddin, Robert Murphy, Caoimhe Hanrahan, Elaine Loughlin, John Ferguson, Conor Judge, Ruairi Waters, Michelle Canavan, Rose Anne Kenny, Martin O’Donnell

**Affiliations:** HRB—Clinical Research Facility, National University of Ireland Galway, Galway D02 V583, Ireland; Galway University Hospital, Newcastle Road, Galway H91 T861, Ireland; Wellcome Trust—HRB, Irish Clinical Academic Training, London NW1 2BE, UK; HRB—Clinical Research Facility, National University of Ireland Galway, Galway D02 V583, Ireland; Galway University Hospital, Newcastle Road, Galway H91 T861, Ireland; HRB—Clinical Research Facility, National University of Ireland Galway, Galway D02 V583, Ireland; Galway University Hospital, Newcastle Road, Galway H91 T861, Ireland; HRB—Clinical Research Facility, National University of Ireland Galway, Galway D02 V583, Ireland; Galway University Hospital, Newcastle Road, Galway H91 T861, Ireland; HRB—Clinical Research Facility, National University of Ireland Galway, Galway D02 V583, Ireland; HRB—Clinical Research Facility, National University of Ireland Galway, Galway D02 V583, Ireland; Galway University Hospital, Newcastle Road, Galway H91 T861, Ireland; HRB—Clinical Research Facility, National University of Ireland Galway, Galway D02 V583, Ireland; Galway University Hospital, Newcastle Road, Galway H91 T861, Ireland; HRB—Clinical Research Facility, National University of Ireland Galway, Galway D02 V583, Ireland; Galway University Hospital, Newcastle Road, Galway H91 T861, Ireland; Mercer's Institute for Successful Ageing (MISA), St James's Hospital, Dublin D08 X9HD, UK; Department of Medical Gerontology, Trinity College Dublin, Dublin 2 D02 PN40, Ireland; HRB—Clinical Research Facility, National University of Ireland Galway, Galway D02 V583, Ireland; Galway University Hospital, Newcastle Road, Galway H91 T861, Ireland

**Keywords:** orthostatic hypotension, hypertension, antihypertensive therapy, systematic review, older people

## Abstract

**Background and purpose:**

Management of antihypertensive therapy is challenging in patients with symptomatic orthostatic hypotension, a population often excluded from randomised controlled trials of antihypertensive therapy. In this systematic review and meta-analysis, we sought to determine whether the association of antihypertensive therapy and adverse events (e.g. falls, syncope), differed among trials that included or excluded patients with orthostatic hypotension.

**Methods:**

We performed a systematic review and meta-analysis of randomised controlled trials comparing blood pressure lowering medications to placebo, or different blood pressure targets on falls or syncope outcomes and cardiovascular events. A random-effects meta-analysis was used to estimate a pooled treatment-effect overall in subgroups of trials that excluded patients with orthostatic hypotension and trials that did not exclude patients with orthostatic hypotension, and tested *P* for interaction. The primary outcome was fall events.

**Results:**

46 trials were included, of which 18 trials excluded orthostatic hypotension and 28 trials did not. The incidence of hypotension was significantly lower in trials that excluded participants with orthostatic hypotension (1.3% versus 6.2%, *P* < 0.001) but not incidences of falls (4.8% versus 8.8%; *P* = 0.40) or syncope (1.5% versus 1.8%; *P* = 0.67). Antihypertensive therapy was not associated with an increased risk of falls in trials that excluded (OR 1.00, 95% CI; 0.89–1.13) or included (OR 1.02, 95% CI; 0.88–1.18) participants with orthostatic hypotension (*P* for interaction = 0.90).

**Conclusions:**

The exclusion of patients with orthostatic hypotension does not appear to affect the relative risk estimates for falls and syncope in antihypertensive trials.

## Key Points

- Co-existing hypertension and orthostatic hypotension is a common management dilemma.
- The exclusion of this cohort from randomised controlled trials of antihypertensive therapy did not alter relative safety effects of treatment.
- The exclusion of this cohort may under-estimate the absolute risk of adverse events such as falls.

## Introduction

Hypertension is a key modifiable risk factor for cardiovascular disease [1–3]. Current guidelines, e.g. European Society of Cardiology/European Society of Hypertension guideline, recommend blood pressure management with a target systolic blood pressure range of 130–139 mm Hg in older adults (≥65 years) [4]. The decision to initiate antihypertensive therapy in older adults, and the choice of blood pressure target in this cohort, requires a trade-off between efficacy and safety (e.g. adverse events such as falls). A particularly challenging cohort of hypertensive patients to manage are those with concomitant orthostatic hypotension. Orthostatic hypotension affects approximately 20% of adults, with prevalence increasing with age, and is an independent risk factor for injurious falls, as well as cardiovascular disease and mortality [5, 6]. Given the prevalence of both conditions and the ageing population [7–9], the overlap of hypertension and orthostatic hypotension is likely to become an increasingly common dilemma for clinicians.

In some randomised controlled trials, individuals with orthostatic hypotension were excluded, either based on clinical history or objective measurement of orthostatic blood pressure measurements. Generalising findings from clinical trials to patients in routine clinical practice requires an understanding of whether such exclusion criteria alter treatment estimates of safety, especially among potentially vulnerable patients.

We sought to determine whether the association of antihypertensive therapy with adverse events, such as falls, fracture or syncope differed by exclusion of participants with orthostatic hypotension in randomised controlled trials of antihypertensive therapy.

## Methods

We performed a systematic review and meta-analysis, adhering to the Cochrane Collaboration Guidelines and the Preferred Reporting Items for Systematic Reviews and Meta-Analyses Guidelines [10, 11]. The meta-analysis was registered with the International Prospective Register of Systematic Reviews (PROSPERO identifier: CRD42022337669).

### Data sources and search strategy

To reduce research waste, we extracted data from a recent meta-analysis of antihypertensive therapy by Albrasi et al., which we considered of sufficiently high quality [12, 13]. We limited our search to dates not included in this review (14 April 2020 onwards). We systematically searched PubMed and Embase databases from 14 April 2020 to 26 May 2022. The search terms included are outlined in the Supplementary Appendix (eMethods I). Two reviewers (CR and RM) screened titles and abstracts using the Rayyan web application [14]. The reference lists of included studies were also reviewed. Full texts of remaining articles (and where applicable clinicaltrials.gov records) were independently assessed by two reviewers (CR and RM), with eligibility based on the pre-determined criteria. Disagreements were resolved by consensus, where a resolution was not reached by discussion, a consensus was reached through a third reviewer (MOD).

### Eligibility criteria

Studies were considered eligible if they were (i) randomised controlled trials, (ii) included adults greater than 18 years, (iii) evaluated antihypertensives compared with placebo, combination of antihypertensive agents compared to fewer antihypertensives, or higher compared to lower blood pressure targets, (iv) reported at least one of the following adverse events; falls, syncope, fracture, orthostatic hypotension or hypotension, and (v) had at least 650 patient years of follow-up. Similar to the prior search by Albasri et al. [13], we specified an a priori limit on patient years of follow-up to ensure that included studies were large enough to accrue outcome events.

### Data extraction

Data were extracted independently by two authors (CR and CH) using a standardised pre-determined data collection form. For each study, we extracted the title, year of publication, follow-up duration, antihypertensive regime/agent/target, intervention and control participant numbers, inclusion/exclusion of orthostatic hypotension (and criterion applied), baseline blood pressure, falls, syncope, fracture, orthostatic hypotension, hypotension events, primary outcome of individual studies, and all-cause mortality. Data were compared for inconsistencies and merged into a pre-final dataset which was checked independently by a third reviewer (RM).

### Outcomes

The primary outcome was fall events. The secondary outcome measures were syncope, fracture, orthostatic hypotension, hypotension, all-cause mortality and original primary outcomes of individual trials. The definition of original primary outcomes differed between the individual trials and are outlined in eTable S1.

### Data synthesis and analysis

A descriptive analysis of trials and baseline characteristics of participants are reported in Table 1. We calculated the odds ratio (OR) and 95% confidence intervals (CI) for each outcome of interest from individual studies. Weighted pooled treatment effects were calculated overall and individually for trials that excluded patients with orthostatic hypotension/symptomatic hypotension, and trials that did not exclude this population, using restricted maximum likelihood estimation to fit a random effects meta-analysis model. Our objective was to determine if there was a difference in risk of adverse events by inclusion/exclusion of this population group. We statistically tested for a difference in treatment effect by testing a *P* for interaction between trials that excluded patients with orthostatic hypotension/symptomatic hypotension, and those which did not. *P* for interaction <0.1 was considered evidence of statistical heterogeneity [15]. Trials that excluded patients with orthostatic hypotension (either using an objective blood pressure criterion or a known diagnosis of orthostatic hypotension) and trials that excluded patients with symptomatic hypotension were combined as the rate of orthostatic hypotension in the control group of the latter trials was low (eFigure S1) and, therefore, these criteria likely lead to the exclusion of the majority of participants with orthostatic hypotension, this group are referred to as ‘Excluded OH’ in the results. Risk of bias assessments were performed independently by two reviewers (CR and EL) using the Cochrane risk of bias 2 tool for randomised controlled trials [16]. Publication bias was assessed using a funnel plot. Statistical analysis was performed using the Metafor package on R Statistical Software (Version 3.6.1) [17].

**Table 1:** Characteristics by orthostatic hypotension exclusion

	Orthostatic hypotension exclusion		
Characteristic	No *N* = 28 (61%)^a^	Yes *N* = 18 (39%)^a^	*P*-value^b^
**Age (years)**	64 (7)	66 (6)	0.29
**Female (%)**	37 (14)	35 (15)	0.52
**Follow-up duration (months)**	33 (18)	38 (16)	0.35
Unknown	1	0	
**Population type**			
CVD/stroke/heart failure	17 trials (60.7%)	10 trials (55.6%)	
T2DM/CVD risk factor/hypertension	9 trials (32.1%)	5 trial (27.8%)	
CVD or increased risk of CVD	0 trials	2 trials (11.1%)	
Other	2 trials (7.1%)	2 trial (11.1%)	
**Baseline systolic BP (intervention group)**	137 (14)	138 (11)	0.82
Unknown	3	2	
**Baseline systolic BP (control group)**	137 (15)	139 (11)	0.85
Unknown	3	2	
**Baseline diastolic BP (intervention group)**	79.4 (6.0)	78.8 (3.4)	0.93
Unknown	4	4	
**Baseline diastolic BP (control group)**	79.5 (5.9)	78.9 (3.3)	0.88
Unknown	4	4	

^a^Mean (SD)

^b^Wilcoxon Rank Sum test

## Results

The systematic search of articles published between 14 April 2020 and 26 May 2022 identified 2,042 records. Following title and abstract screening, 14 were considered potentially relevant. After the application of eligibility criteria to full text review of studies included in the prior meta-analysis by Albrasi et al. and potentially relevant studies identified in the updated search, 46 trials (*n* = 233,357) were included (eFigure S2). The 46 trials had a mean follow-up duration of 34.9 months, and included 18 trials (*n* = 97,976) that excluded those with orthostatic hypotension and 28 trials (*n* = 135,381) that did not exclude those with orthostatic hypotension. Characteristics of trials and participants by OH exclusion categories are outlined in Table 1. There was no difference in the mean age of participants by OH exclusion categories. The baseline systolic BP and diastolic BP were similar in both groups. Characteristics of individual trials are outlined in Table 2.

**Table 2:** Individual trial characteristics

Trial name	Year	Trial population	Intervention	Control	Intervention baseline SBP	Control baseline SBP
**Excluded those with OH**						
NILVAD [18]	2018	Aged >50, probable Alzheimers disease with MMSE ≥12 and < 27	Nilvadipine	Placebo	138	137
HOPE-3 [33]	2016	Men >55 years and women >65 years with one CVD risk factor	Candesartan and hydrochlorthiazide	Placebo	138.2	137.9
SPRINT [19]	2015	>50 years, SBP 130–180 mm Hg with increased CVD risk, no diabetes	SBP target <120 mm Hg	SBP target <140 mm Hg	139.7	139.7
ORIENT [31]	2011	Type 2 diabetes with poor renal function	Olmesartan	Placebo	141.7	140.8
EMPHASIS-HF [20]	2011	NYHA class II heart failure	Eplerenone	Placebo	124	124
GISSI-AF [48]	2009	Atrial fibrillation and underlying CVD	Valsartan	Placebo	138.2	139
SANDS [32]	2009	Native Americans with type 2 diabetes	BP target ≤115/75 mm Hg	BP target ≤130/80 mm Hg	128.7	132.6
ONTARGET [22]	2008	Existing vascular disease or diabetes	Ramipril or telmisartan	Ramipril+ telmisartan combination	141.7	141.9
HYVET Trial [45, 76]	2008	>80 years with hypertension	Indapamide and/or perindopril	Placebo	173	173
TRANSCEND [21]	2008	CVD or diabetes with end organ damage	Telmisartan	Placebo	140.7	141.3
PEACE [35]	2004	Myocardial infarction or bypass in past 3 months	Trandolapril	Placebo	134	133
CHARM-Preserved [50]	2003	NYHA class II-IV heart failure	Candesartan	Placebo	136	136.3
CHARM-ADDED [47]	2003	NYHA class II-IV heart failure	Candesartan	Placebo	124.7	125.6
CHARM-Alternative [46]	2003	Heart failure	Candesartan	Placebo	129.9	130.3
EUROPA [49]	2003	Stable coronary heart disease without heart failure	Perindopril	Placebo	137	137
PROGRESS [51]	2001	Previous stroke or transient ischaemic attack	Perindopril and indapamide	Placebo	147	147
Multicentre Diltiazem Postinfarction Trial [52]	1988	Admitted to hospital with acute myocardial infarction	Diltiazem	Placebo		
The Norwegian Multicenter Study [34]	1981	Admitted to hospital with acute myocardial infarction	Timolol	Placebo		
**OH not excluded**						
STEP [36]	2021	60–80 years, Han ethnicity with hypertension with a systolic blood pressure 140–190 mm Hg	SBP target 110– < 130 mm Hg	SBP target 130– < 150 mm Hg	146.1	146
INFINITY [23]	2019	>75 years hypertension	SBP target ≤130 mm Hg	SBP target ≤145 mm Hg	149.7	152
Intensive Antihypertensive Treatment for Elderly [44]	2013	>70 years with hypertension	BP target ≤140/90 mm Hg	BP target <150/90 mm Hg	158.8	160.3
SPS3 [29]	2013	Stroke within past 6 months	SBP target <130 mm Hg	SBP target 130–149 mm Hg	142	144
ALTITUDE [24]	2012	Type 2 diabetes	Aliskiren	Placebo	137.3	137.3
ASPIRE [25]	2011	Post-myocardial infarction	Aliskiren	Placebo	121.6	121.7
ROADMAP Trial [53]	2011	Type 2 diabetes	Olmesartan	Placebo	137	136
ACCORD [27, 37]	2010	Type 2 diabetes at high risk CVD	BP target <120 mm Hg	BP target <140 mm Hg	139	139.4
NAVIGATOR [26]	2010	Type 2 diabetes	Valsartan	Placebo	139.4	139.9
I-PRESERVE [38]	2008	Heart failure	Irbesartan	Placebo	137	136
PRoFESS [28]	2008	>55 years and ischaemic stroke	Telmisartan	Placebo	144.1	144.2
TROPHY [39]	2006	Pre-hypertensive population	Candesartan	Placebo	133.9	134.1
SENIORS [64]	2005	>70 years with heart failure	Nebivolol	Placebo	138.6	139.5
NICOLE [55]	2003	<75 years and previous angioplasty	Nisoldipine	Placebo		
VALIANT [54]	2003	Myocardial infarction with left ventricular systolic dysfunction	Valsartan and captopril	Valsartan or captopril	122.7	122.5
AASK [40]	2002	African-Americans with hypertension and renal disease	MAP ≤92 mm Hg	MAP 102–107 mm Hg	152	149
BEST [41]	2001	NYHA class III or heart failure	Bucindolol	Placebo	117	117
Val-HeFT [56]	2001	Heart failure	Valsartan	Placebo	123	124
HOPE Trial [58]	2000	>55 years, high CVD risk	Ramipril	Placebo	139	139
MERIT-HF [57]	2000	NYHA class II-IV heart failure	Metoprolol	Placebo		
CCS-I [59]	1997	Acute myocardial infarction	Captopril	Placebo	127	126
MACB [61]	1995	Referred for coronary artery bypass grafting	Metoprolol	Placebo	120	120
TRACE [60]	1995	Admitted to hospital with acute myocardial infarction	Trandolapril	Placebo	122	120
AIRE [42]	1993	Acute myocardial infarction and evidence of heart failure	Ramipril	Placebo		
Dutch TIA Trial [62]	1993	Previous TIA	Atenolol	Placebo	157	158
CONSENSUS II [63]	1992	Post-myocardial infarction	Enalapril	Placebo	133	134
SHEP [30]	1991	>60 years with Isolated Systolic Hypertension	Chlorthalidone with or without atenolol (or reserpine if atenolol contraindicated)	Placebo	170.5	170.1
BHAT [43]	1982	Admitted to hospital with acute myocardial infarction	Propranolol	Placebo	112.3	111.7

### Antihypertensive treatment and falls events

**Figure 1:**
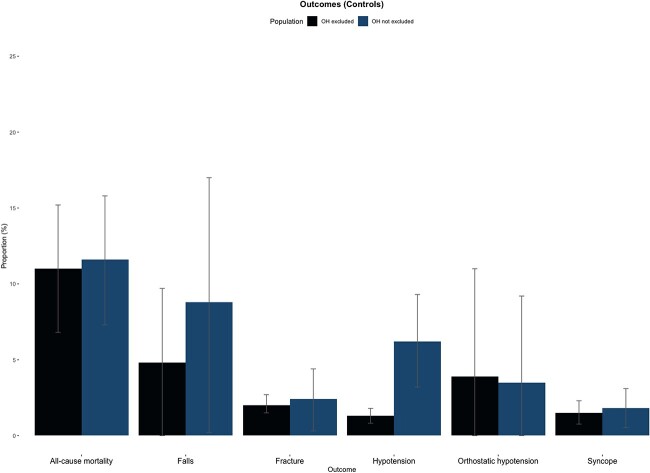
Event rates in the control group. Bar chart depicting the incidence rates of outcomes within the control group. The black column represents trials that excluded those with orthostatic hypotension, and the blue column represents trials that did not exclude participants with orthostatic hypotension. The *y*-axis represents the percentage of trial population.

**Figure 2:**
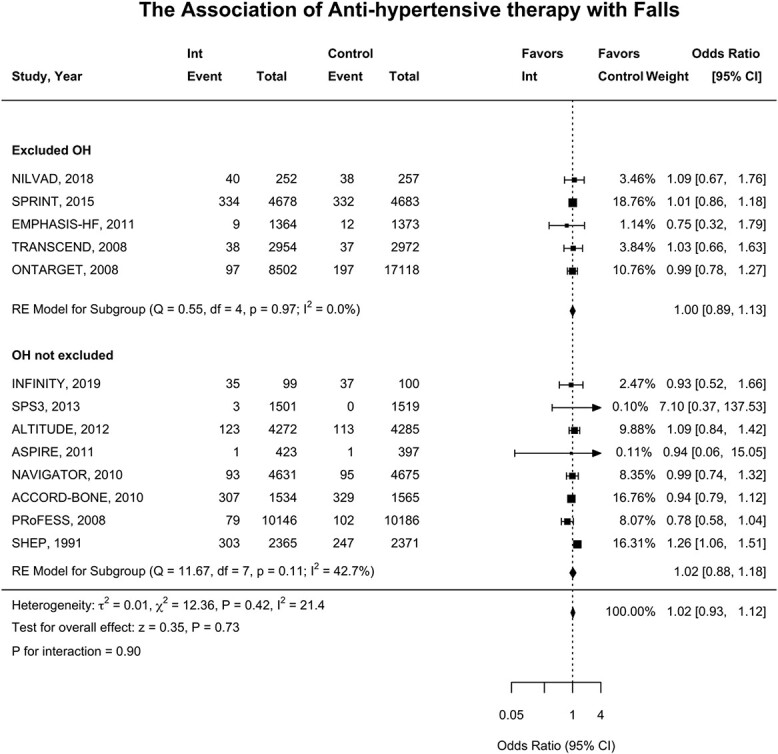
The association of antihypertensive therapy with falls. Forest plot demonstrates the association of antihypertensive therapy and falls events. The squares and bars represent the mean values and 95% confidence intervals of the effect sizes, whereas the area of the squares reflects the weight of the studies. The combined effects appear as diamonds and the vertical dashed line represents the line of no effect. Int, intervention; CI, confidence interval.

13 trials (*n* = 94,222) reported falls, and there were 3,002 falls events during the follow-up [18–30]. The baseline incidence of falls in the control group was 4.8% in trials that excluded orthostatic hypotension compared with 8.8% in trials which did not exclude participants with orthostatic hypotension (*P*-value = 0.40) (Figure 1). The association of antihypertensive treatment and falls was similar for trials that excluded those with orthostatic hypotension (OR 1.00; 95% CI, 0.89–1.13) and trials which did not exclude those with orthostatic hypotension (OR, 1.02; 95% CI, 0.88–1.18) (*P*-interaction = 0.90) (Figure 2).

### Antihypertensive treatment and syncope events

**Figure 3:**
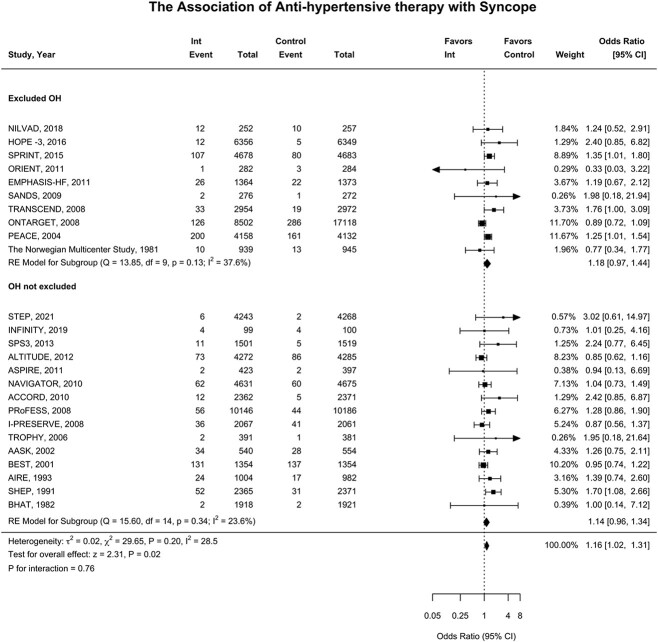
The association of antihypertensive therapy with syncope. Forest plot demonstrates the association of antihypertensive therapy and syncope events. The squares and bars represent the mean values and 95% confidence intervals of the effect sizes, whereas the area of the squares reflects the weight of the studies. The combined effects appear as diamonds and the vertical dashed line represents the line of no effect. Int, intervention; CI, confidence interval.

26 trials (*n* = 145,986) reported syncope, and there were 2,101 syncope events during the follow-up [18–26, 28–43]. The baseline incidence of syncope in the control group was 1.5% in trials that excluded orthostatic hypotension compared to 1.8% in trials which did not exclude participants with orthostatic hypotension (*P*-value = 0.67) (Figure 1). The association of antihypertensive treatment and syncope was similar for trials that excluded those with orthostatic hypotension (OR, 1.18; 95% CI, 0.97–1.44) and trials which did not exclude those with orthostatic hypotension (OR, 1.14; 95% CI, 0.96–1.34) (*P*-interaction = 0.76) (Figure 3).

### Antihypertensive treatment and fracture events

15 trials (*n* = 103,564) reported fracture, and there were 2,134 fracture events during the follow-up [18, 20–22, 25–28, 30, 31, 33, 36, 38, 44, 45]. The baseline incidence of fracture in the control group was 2.0% in trials that excluded orthostatic hypotension compared to 2.4% in trials which did not exclude participants with orthostatic hypotension (*P*-value = 0.78) (Figure 1). The association of antihypertensive treatment and fracture was similar for trials that excluded those with orthostatic hypotension (OR, 1.00; 95% CI, 0.85–1.17) and trials which did not exclude those with orthostatic hypotension (OR, 0.96; 95% CI, 0.81–1.14) (*P*-interaction = 0.76) (eFigure S3).

### Antihypertensive treatment and orthostatic hypotension

Eight trials (*n* = 59,603) reported orthostatic hypotension, and there were 2,071 orthostatic hypotension events during the follow-up [19–22, 24, 31, 38, 41]. The baseline incidence of orthostatic hypotension in the control group was 3.9% in trials that excluded orthostatic hypotension compared to 3.5% in trials which did not exclude participants with orthostatic hypotension (*P*-value = 0.91) (Figure 1). The association of antihypertensive treatment and orthostatic hypotension was similar for trials that excluded those with orthostatic hypotension (OR, 0.93; 95% CI, 0.73–1.18) and trials which did not exclude those with orthostatic hypotension (OR, 1.11; 95% CI, 0.90–1.37) (*P*-interaction = 0.26) (eFigure S4).

### Antihypertensive treatment and hypotension

37 trials (*n* = 202,080) reported hypotension, and there were 12,363 hypotension events during the follow-up [18–22, 25, 26, 28, 29, 31, 32, 34, 36–38, 41–43, 46–64]. The baseline incidence of hypotension in the control group was 1.3% in trials that excluded orthostatic hypotension compared to 6.2% in trials which did not exclude participants with orthostatic hypotension (*P*-value = <0.01) (Figure 1). The association of antihypertensive treatment and hypotension was similar for trials that excluded those with orthostatic hypotension (OR, 1.91; 95% CI, 1.58–2.31) and trials which did not exclude those with orthostatic hypotension (OR, 1.73; 95% CI, 1.42–2.11) (*P*-interaction = 0.47) (eFigure S5).

### Antihypertensive treatment and all-cause mortality

40 trials (*n* = 206,463) reported mortality, and there were 21,771 all-cause mortality events during the follow-up [19–22, 24–26, 28, 31–34, 36–39, 41–43, 45–64]. The baseline incidence of mortality events in the control group was 11.0% in trials that excluded orthostatic hypotension compared to 11.6% in trials which did not exclude participants with orthostatic hypotension (*P*-value = 0.85) (Figure 1). The association of antihypertensive treatment and hypotension was similar for trials that excluded those with orthostatic hypotension (OR, 0.90; 95% CI, 0.83–0.97) and trials which did not exclude those with orthostatic hypotension (OR, 0.92; 95% CI, 0.86–0.98) (*P*-interaction = 0.57) (eFigure S6).

### Antihypertensive treatment and original primary outcomes reported in trial

Among 40 trials (*n* = 229,585), which reported a dichotomous primary outcome, there were 33,882 events during the follow-up [19–22, 24, 26, 28–31, 33–39, 41–52, 54–64]. Definitions of primary outcomes varied between trials (eTable S1). The association of antihypertensive treatment and the primary outcome was similar for trials that excluded those with orthostatic hypotension (OR, 0.83; 95% CI, 0.77–0.89) and trials which did not exclude those with orthostatic hypotension (OR, 0.85; 95% CI, 0.80–0.91) (*P*-interaction = 0.55) (eFigure S7).

### Risk of bias

The risk of bias was assessed for 46 trials (eFigure S8). It was deemed to be ‘low’ in 38 trials, ‘some concerns’ in 7 trials and ‘high risk’ in 1 trial. The randomisation lead to concerns for three trials [19, 34, 58], missing outcome data, which lead to concerns, for one trial [43] and selection of the reported result for four trials [44, 61–63]. Publication bias was assessed using contour enhanced funnel plots, which were symmetrical (eFigure S9a and b).

## Discussion

In this systematic review and meta-analysis, we found no significant difference in the odds of adverse events including falls, syncope, fracture or hypotension associated with antihypertensive therapy between groups by orthostatic hypotension exclusion. However, the event rates of hypotension were significantly lower in trials that excluded participants with orthostatic hypotension (*P*-value ≤0.01). The event rates of falls and fracture were also numerically lower in trials that excluded participants with orthostatic hypotension, although the difference was not statistically significant.

In 2019, the Global Burden of Disease collaboration estimated that age-standardised prevalence rates of hypertension were 32% for women and 34% for men aged 30–79 years [7]. Hypertension is a leading risk factor for cardiovascular disease, stroke, dementia and mortality [2, 65–67]. Previous meta-analyses have shown that blood pressure lowering is associated with a reduced risk of cardiovascular disease, mortality and dementia [68, 69]. Orthostatic hypotension has also been reported to be an independent risk factor for cardiovascular disease and mortality [5]. It is estimated that approximately 10% of hypertensive individuals attending specialist clinics have co-existing orthostatic hypotension [70, 71]. Clinicians may be more cautious initiating antihypertensive therapy in those with co-existing orthostatic hypotension; however, the risk of orthostatic hypotension is higher in those with uncontrolled hypertension [72, 73]. Blood pressure management in those with both supine hypertension and orthostatic hypotension is a common and challenging dilemma for clinicians and patients.

Based on the results of randomised controlled trials, such as SPRINT, lower blood pressure targets have been recommended to reduce cardiovascular risk. However, these guidelines suggest caution in older adults with multimorbidity and advise that clinicians consider the risk and benefits of a particular therapy or target and tailor the decision to the individual patient [74]. As highlighted in our study, a large proportion of clinical trials evaluating blood pressure lowering excluded participants with orthostatic hypotension. Exclusion criterion are applied to clinical trial recruitment on the basis of medical conditions, such as orthostatic hypotension, where the condition may pose an increased risk of adverse events which may compromise the safety of the participant. However, where this comorbidity commonly co-exists with the condition of interest, e.g. hypertension, the impact of this exclusion criterion on the external validity of results should be considered. The exclusion of this cohort from clinical trials of antihypertensive therapy poses challenges to shared decision making where the true risk-benefit of an intervention or blood pressure target is not known, rather extrapolated from a different population cohort.

### Limitations of our study

This study has a number of limitations. First, the definition of orthostatic hypotension differed between trials with some studies (e.g. SPRINT) employing a systematic exclusion criterion at screening, whereas other studies excluded participants based on a patient-reported history of orthostatic hypotension. Both methods may be prone to misclassification, given the challenges in reproducibility of blood pressure measurements in orthostatic hypotension [75]. Trials that excluded patients due to symptomatic hypotension were combined with trials that excluded patients with orthostatic hypotension, due to the low incidence of orthostatic hypotension in these trials. Secondly, as this review focused on adverse events, findings may be confounded by the selective outcome reporting bias. Thirdly, we limited our search to large randomised controlled trials that reported pre-specified adverse events. Finally, this study did not include patient and public involvement that may have provided valuable insights.

## Conclusion

Exclusion of patients with orthostatic hypotension from antihypertensive trials did not alter relative effects of treatment, with respect to falls or syncope. However, clinicians should be cognisant when counselling individuals with orthostatic hypotension and supine hypertension, regarding the potential side-effects of antihypertensive therapy, that trials which excluded those with orthostatic hypotension may under-estimate the absolute risk of adverse effects such as symptomatic hypotension and possibly falls/syncope, as event rates were lower in trials that excluded this population.

## Supplementary Material

aa-22-1470-File002_afad044

## Data Availability

The data that support the findings of this study are available from the corresponding author upon reasonable request.
