# Supplementary material for: Randomised controlled trials of antihypertensive therapy: does exclusion of orthostatic hypotension alter treatment effect? A systematic review and meta-analysis
Source: Age Ageing. 2023 Apr 1;52(4):afad044. doi: 10.1093/ageing/afad044 (PMC10883139; doi:10.1093/ageing/afad044)
Supplement: aa-22-1470-File002_afad044 [file aa-22-1470-file002_afad044.zip › aa-22-1470-File002_afad044.docx]

**Randomised controlled trials of antihypertensive therapy: does exclusion of orthostatic hypotension alter treatment effect?**

**A systematic review and meta-analysis**

**Supplementary Appendix**

eMethods 1 – Search Strategy

Studies were searched from 20^th^ April, 2020 to May 26th, 2022.

Search terms were as follows:

hypertension *OR* hypertension, malignant *OR* hypertension, renal *OR* hypertension, renovascular *OR* hypertens* *OR* antihypertens* *OR* anti-hypertens* *OR* blood pressure *OR* diastole *OR* systole *OR* BP *OR* DBP *OR* SBP

OR

antihypertensive agents OR acebutolol OR alprenolol OR amlodipine OR atenolol OR bendroflumethiazide OR bepridil OR betaxolol OR bethanidine OR bisoprolol OR bupranolol OR captopril OR carteolol OR celiprolol OR chlORisondamine OR chlorothiazide OR chlorthalidone OR cilazapril OR clonidine OR cyclopenthiazide OR diazoxide OR dihydralazine OR diltiazem OR doxazosin OR enalapril OR enalaprilat OR felodipine OR fosinopril OR guanabenz OR hydralazine OR hydrochlorothiazide OR hydroflumethiazide OR indapamide OR indORamin OR isradipine OR labetalol OR lisinopril OR losartan OR methyldopa OR metipranolol OR metolazone OR metoprolol OR mibefradil OR minoxidil OR nadolol OR nicardipine OR nimodipine OR nisoldipine OR nitrendipine OR oxprenolol OR pempidine OR penbutolol OR perindopril OR pinacidil OR pindolol OR polythiazide OR prazosin OR propranolol OR ramipril OR timolol OR todralazine OR trichlormethiazide OR xipamide OR antihypertensive OR anti- hypertensive

OR

adrenergic alpha-antagonists OR adrenergic alpha-1 receptor antagonists OR doxazosin OR indORamin OR labetalol OR prazosin OR adrenergic alpha-2 receptor antagonists OR adrenergic beta-antagonists OR alprenolol OR bupranolol OR carteolol OR dihydroalprenolol OR metipranolol OR nadolol OR oxprenolol OR penbutolol OR pindolol OR propranolol OR sotalol OR timolol OR adrenergic beta-1 receptor antagonists OR acebutolol OR atenolol OR betaxolol OR bisoprolol OR celiprolol OR metoprolol OR adrenergic beta-2 receptor antagonists OR adrenergic beta-3 receptor antagonists OR adrenergic alpha-antagonist OR adrenergic alphaantagonist

OR

angiotensin-converting enzyme inhibitors OR captopril OR cilazapril OR enalapril OR enalaprilat OR fosinopril OR lisinopril OR perindopril OR ramipril OR angiotensin- converting enzyme inhibitor

OR

angiotensin receptor antagonists OR angiotensin ii type 1 receptor blockers OR losartan OR saralasin OR angiotensin ii type 2 receptor blockers OR angiotensin receptor antagonist

OR

calcium channel blockers OR amlodipine OR amrinone OR bencyclane OR bepridil OR diltiazem OR felodipine OR fendiline OR flunarizine OR gallopamil OR isradipine OR lidoflazine OR mibefradil OR nicardipine OR nifedipine OR nimodipine OR nisoldipine OR nitrendipine OR tiapamil hydrochloride OR verapamil OR calcium channel blocker

OR

diuretics OR amiloride OR bendroflumethiazide OR chlorthalidone OR cyclopenthiazide OR furosemide OR hydrochlORothiazide OR hydroflumethiazide OR indapamide OR mefruside OR methazolamide OR methyclothiazide OR metolazone OR polythiazide OR spironolactone OR ticrynafen OR triamterene OR trichlormethiazide OR xipamide OR diuretics, osmotic OR diuretics, potassium sparing OR epithelial sodium channel blockers OR mineralocorticoid receptor antagonists OR sodium chloride symporter inhibitors OR sodium potassium chloride symporter inhibitors OR diuretic

OR

vasodilator agents OR bencyclane OR bepridil OR celiprolol OR chromonar OR colforsin OR cromakalim OR cyclandelate OR diazoxide OR dilazep OR diltiazem OR enoximone OR ergoloid mesylates OR erythritol OR flunarizine OR iloprost OR isradipine OR lidoflazine OR minoxidil OR molsidomine OR nicardipine OR nicergoline OR nifedipine OR nimodipine OR nisoldipine OR nitrendipine OR nylidrin OR oxprenolol OR oxyfedrine OR perhexiline OR phenoxybenzamine OR pinacidil OR pindolol OR prenylamine OR s-nitrosoglutathione OR suloctidil OR trapidil OR trimetazidine OR verapamil OR vasodilator

OR

Aldosterone

OR

Chlorisondamine

OR

Mineralocorticoids OR Desoxycorticosterone OR Desoxycorticosterone Acetate

OR
Pempidine

OR

Renin-Angiotensin System

OR

accupro OR accuretic OR acebutolol OR acepril OR acezide OR adalat OR adanif OR adcirca OR adipine OR adizem OR aldomet OR aliskiren OR alphavase OR ambrisentan OR amias OR amiloride OR amlodipine OR amlostin OR angilol OR angiopine OR angiozem OR angitil OR antipressan OR apresoline OR aprinox OR aprovel OR apsolox OR atenamin OR atenix OR atenolol OR baratol OR baycaron OR bedranol OR bendroflumethiazide OR benthiazide OR berkatens OR berkolol OR berkozide OR beta-adalat OR beta-cardone OR betadur OR betaloc OR beta-progane OR bi-carzem OR bipranix OR bisoprolol OR blocadren OR bosentan OR brevibloc OR britazim OR cabren OR calanif OR calazem OR calchan OR calcicard OR calcilat OR candesartan OR capoten OR capozide OR capto-co OR captomex OR captopril OR captopril OR caracace OR carace OR cardene OR cardicor OR cardide OR cardilate OR cardioplen OR cardozin OR cardura OR carvedilol OR cascor OR catapres OR celectol OR celiprolol OR centyl OR chlORothaizide OR chlortalidone OR cilazapril OR ciolixil OR clonidine OR clopamide OR co-amilozide OR coaprovel OR co-betaloc OR co-diovan OR congescor OR co-prenozide OR coracten OR cordilox OR corgard OR corgaretic OR coroday OR co-tenidone OR co-tenidone OR coversyl OR cozaar OR co-zidocapt OR cyclopenthiazide OR cyclopenthiazide OR delvas OR diazoxide OR dibenyline OR dilcardia OR diltiazem OR dilzem OR diovan OR disogram OR diurexan OR diuril OR dopamet OR doxadura OR doxazosin OR ecopace OR ednyt OR emcOR OR enalapril OR enalpril OR enduron OR eprosartan OR esidrex OR esmolol OR ethibide OR ethimil OR eucardic OR exforge OR felendil OR felodipine OR felogen OR felotens OR fortipine OR fosinopril OR genalat OR gopten OR horizem OR hydralazine OR hydrenox OR hydrochlorothiazide OR hydroflumethiazide OR hydromet OR hygroton OR hypertene OR hypolar OR hypovase OR hytrin OR iloprost OR imidapril OR indapamide OR inderal OR indipam OR indoramin OR innovace OR innozide OR irbesartan OR isradipine OR istin OR kalspare OR kalten OR kaplon OR keloc OR kenzem OR labetalol OR labrocol OR lacidipine OR larbex OR lercanidipine OR lisinopril OR lisopress OR loniten OR lopace OR lopranol OR lopresor OR lopresORetic OR lopresoteric OR losartan OR mapemid OR mefruside OR mepranix OR meprobamate OR metalpha OR metenix OR methyclothiazide OR methyldopa OR metolazone OR metoprolol OR metoros OR metroprolol OR micardis OR minoxidil OR moducren OR moduretic OR moexipril OR monocor OR motens OR moxonidine OR nadolol OR natramid OR natrilix OR navidrex OR nebivolol OR neo-bendromax OR neofel OR neo- naclex OR neozipine OR nephril OR nicardipine OR nifedipine OR nifedipress OR nifelease OR nimodrel OR nimotop OR nindaxa OR nivaten OR normetic OR noyada OR odrik OR olmesartan OR olmetec OR optil OR opumide OR oxprenolol OR parmid OR perdix OR perindopril OR perinodopril OR phenoxybenamine OR phentolamine OR physiotens OR pindolol OR pinodolod OR plendil OR pralenal OR prazosin OR prescal OR prestim OR probeta OR propanix OR propranolol OR quinapril OR quinil OR ramipril OR ranvera OR rapORsin OR rapranol OR rasilez OR rawel OR retalzem OR revatio OR rogitine OR saluric OR secadrex OR sectral OR securon OR sevikar OR sildenafil OR slocinx OR slofedipine OR slo- pro OR slopronol OR slow-pren OR slozem OR sodium nitroprusside OR sotacor OR sotalol

OR spiro-co spironolactone OR spiroprop OR staril OR syprol OR tadalafil OR tanatril OR tarka OR telmisartan OR tenavoid OR tenben OR tenchlor OR tenif OR tenoret OR tenoretic OR tenormin OR tensaid OR tensipine OR tensopril OR terazosin OR teveten OR tildiem OR timolol OR tolerzide OR totamol OR totaretic OR tracleer OR trandate OR trandolapril OR trasicor OR trasidrex OR triamterene OR triapin OR tritace OR uard OR unipine OR univer OR valasartan OR valni OR varbim OR vasaten OR vascace OR vascalpha OR ventavis OR verapamil OR verapress OR vera-til OR vertab OR viazem OR visken OR vivacor OR xipamide OR xuret OR zanidip OR zaroxolyn OR zemret OR zemtard OR zestoretic OR zestril OR zida-co zildil OR zolvera

AND

Syncope *OR* fall *OR* injurious fall *OR* injury *OR* hypotension *OR* symptomatic hypotension *OR* orthostatic hypotension *OR* postural hypotension

AND

Randomised controlled trial *OR* Randomised controlled trial *OR* Controlled clinical trial *OR* Randomised *OR* Randomised OR placebo *OR* Drug therapy *OR* Randomly *OR* Trial *OR* Groups

NOT

Animals *NOT* Humans

The search was conducted through Pubmed and EMBASE.

eFigure 1- Event proportion in control group


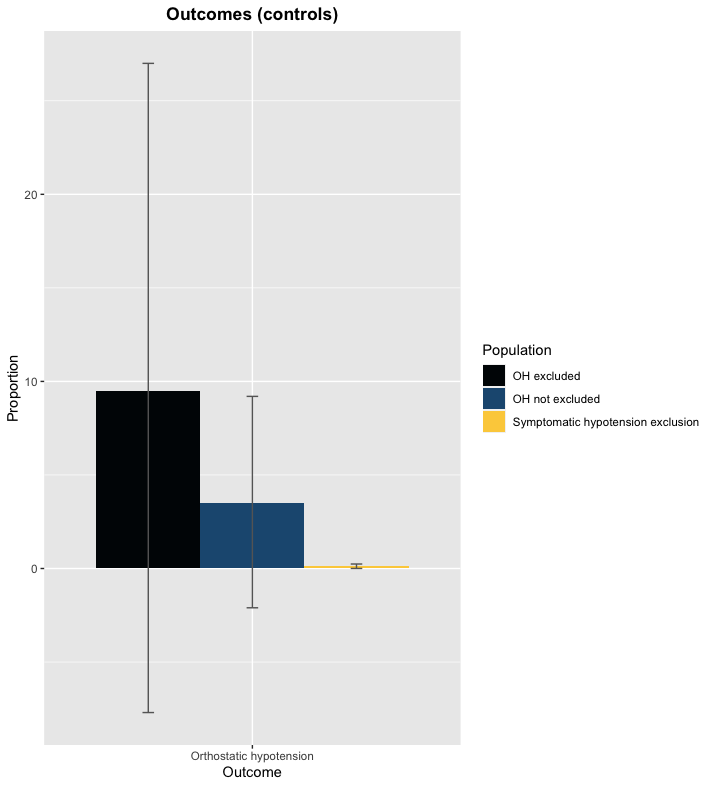


eFigure 2 – PRISMA flow

Records identified from previous systematic review with comprehensive search
(n = 58)

Records identified through database searching
(n = 2042)

Identification

Studies included in qualitative synthesis
(n = 45)

Screening

Reason for exclusion:

Did not report outcome of interest (n=13)

Records excluded
(n = 2023)

Records screened
(n = 2037)

Full-text articles assessed for eligibility
(n =58)

Full-text articles assessed for eligibility
(n =14)

Studies included in qualitative synthesis
(n= 1)

Records after duplicates removed
(n =2037 )

Studies included included

(n = 46)

Included

Eligibility

eFigure 3 – The Association of Anti-hypertensive therapy with Fracture


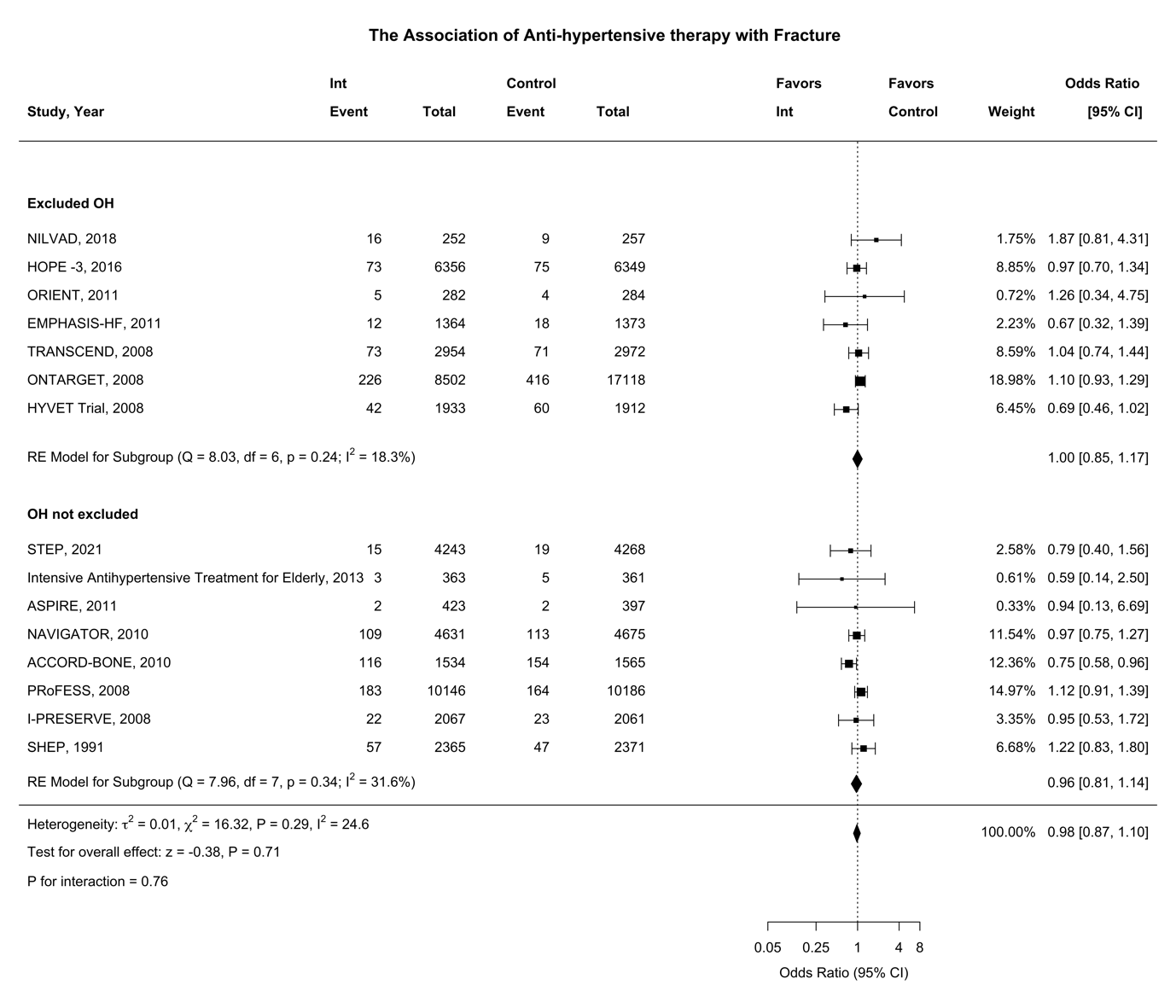


eFigure 3 – Forest plot demonstrating the association of anti-hypertensive therapy and fracture events. The squares and bars represent the mean values and 95% confidence intervals of the effect sizes, while the area of the squares reflects the weight of the studies. The combined effects appear as diamonds and the vertical dashed line represents the line of no effect.

Int-Intervention, CI-Confidence Interval.

eFigure 4 – The Association of Anti-hypertensive therapy with Orthostatic Hypotension
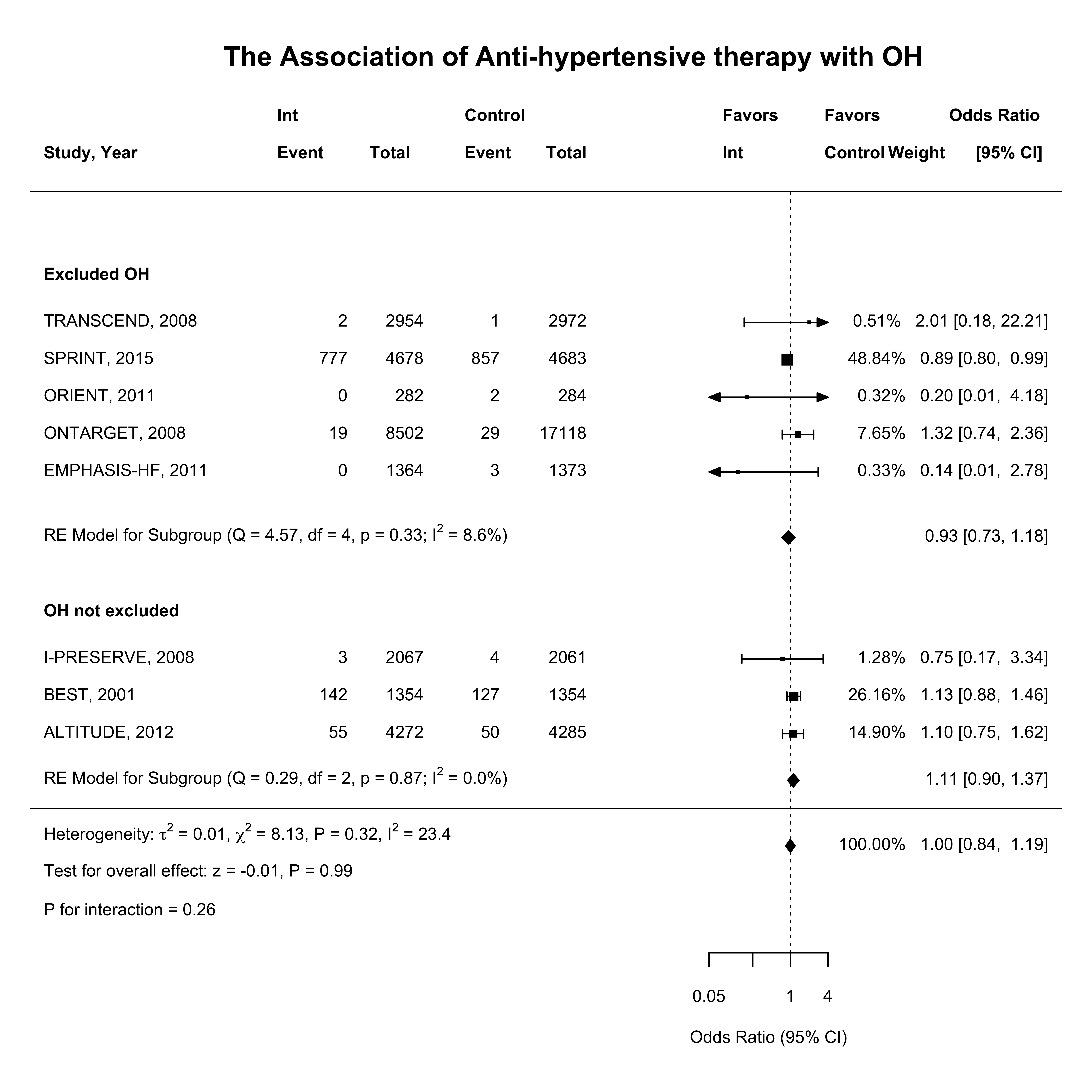


eFigure 4 – Forest plot demonstrating the association of anti-hypertensive therapy and orthostatic hypotension. The squares and bars represent the mean values and 95% confidence intervals of the effect sizes, while the area of the squares reflects the weight of the studies. The combined effects appear as diamonds and the vertical dashed line represents the line of no effect.

Int-Intervention, CI-Confidence Interval, OH-Orthostatic hypotension

eFigure 5 – The Association of Anti-hypertensive therapy with Hypotension


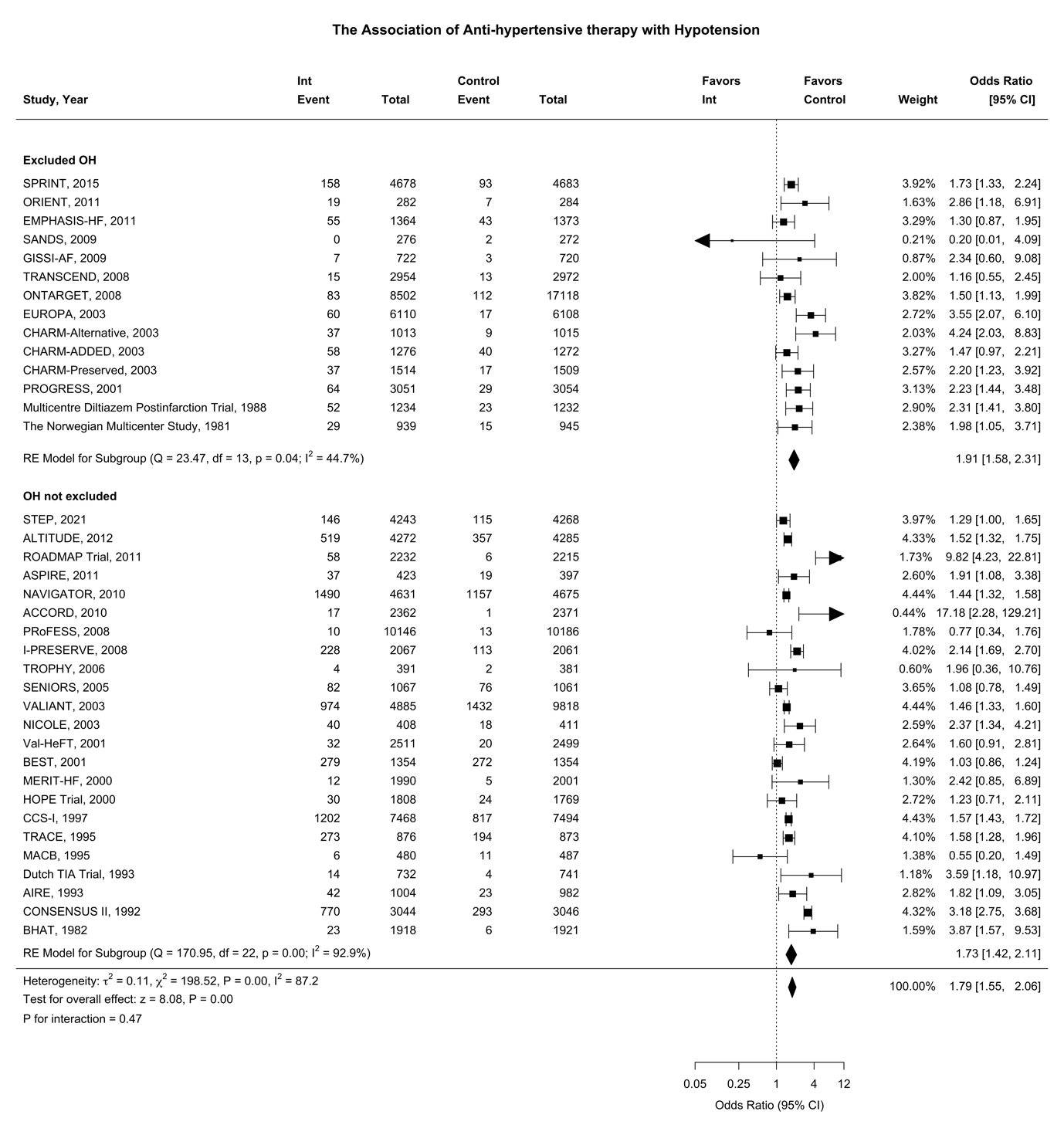


eFigure 5 – Forest plot demonstrating the association of anti-hypertensive therapy and hypotension. The squares and bars represent the mean values and 95% confidence intervals of the effect sizes, while the area of the squares reflects the weight of the studies. The combined effects appear as diamonds and the vertical dashed line represents the line of no effect.

Int-Intervention, CI-Confidence Interval, OH-Orthostatic hypotension

eFigure 6 – The Association of Anti-hypertensive therapy with All-Cause Mortality


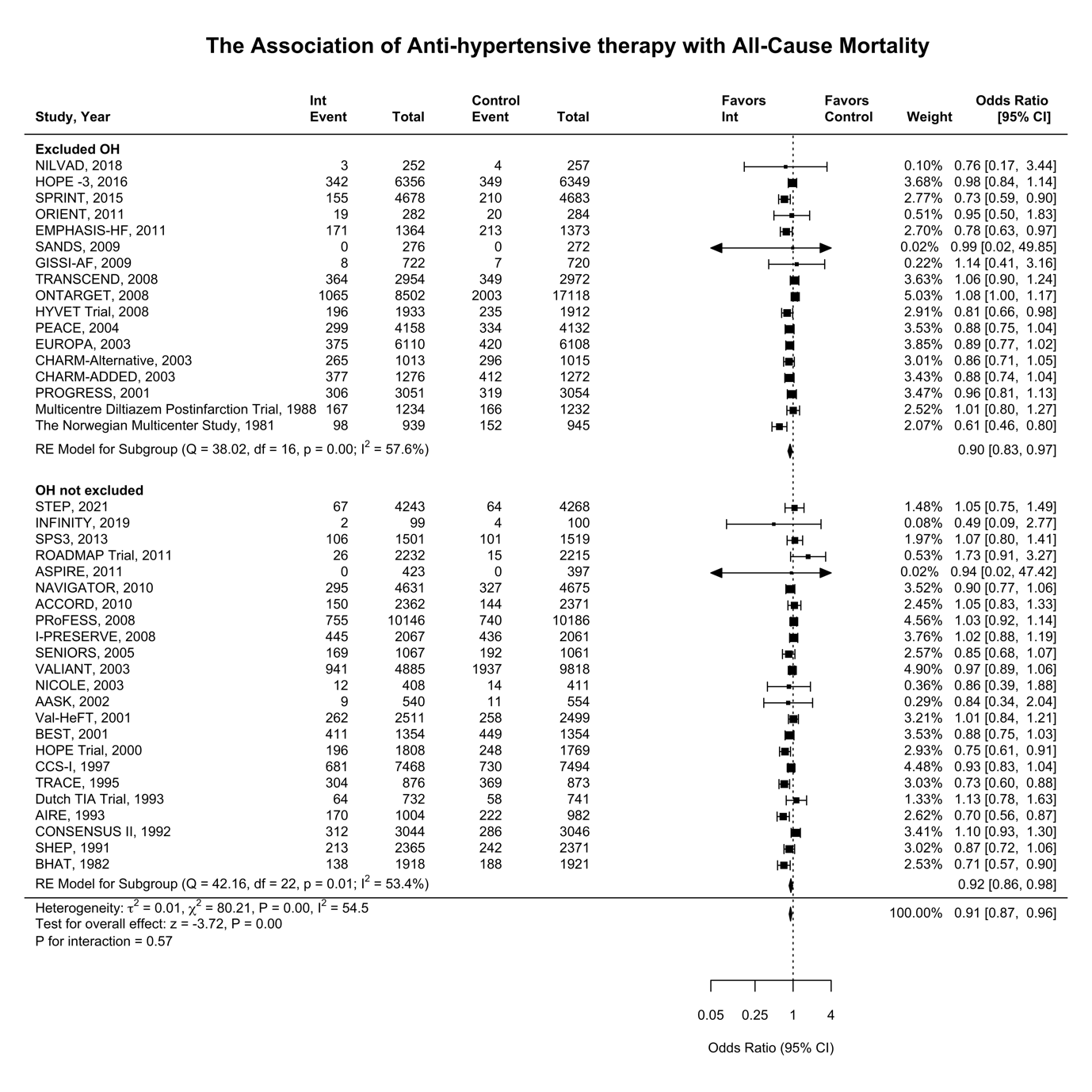


eFigure 6 – Forest plot demonstrating the association of anti-hypertensive therapy and all-cause mortality. The squares and bars represent the mean values and 95% confidence intervals of the effect sizes, while the area of the squares reflects the weight of the studies. The combined effects appear as diamonds and the vertical dashed line represents the line of no effect. Int-Intervention, CI-Confidence Interval, OH-Orthostatic hypotension

eFigure 7 – The Association of Anti-hypertensive therapy with Primary Outcome


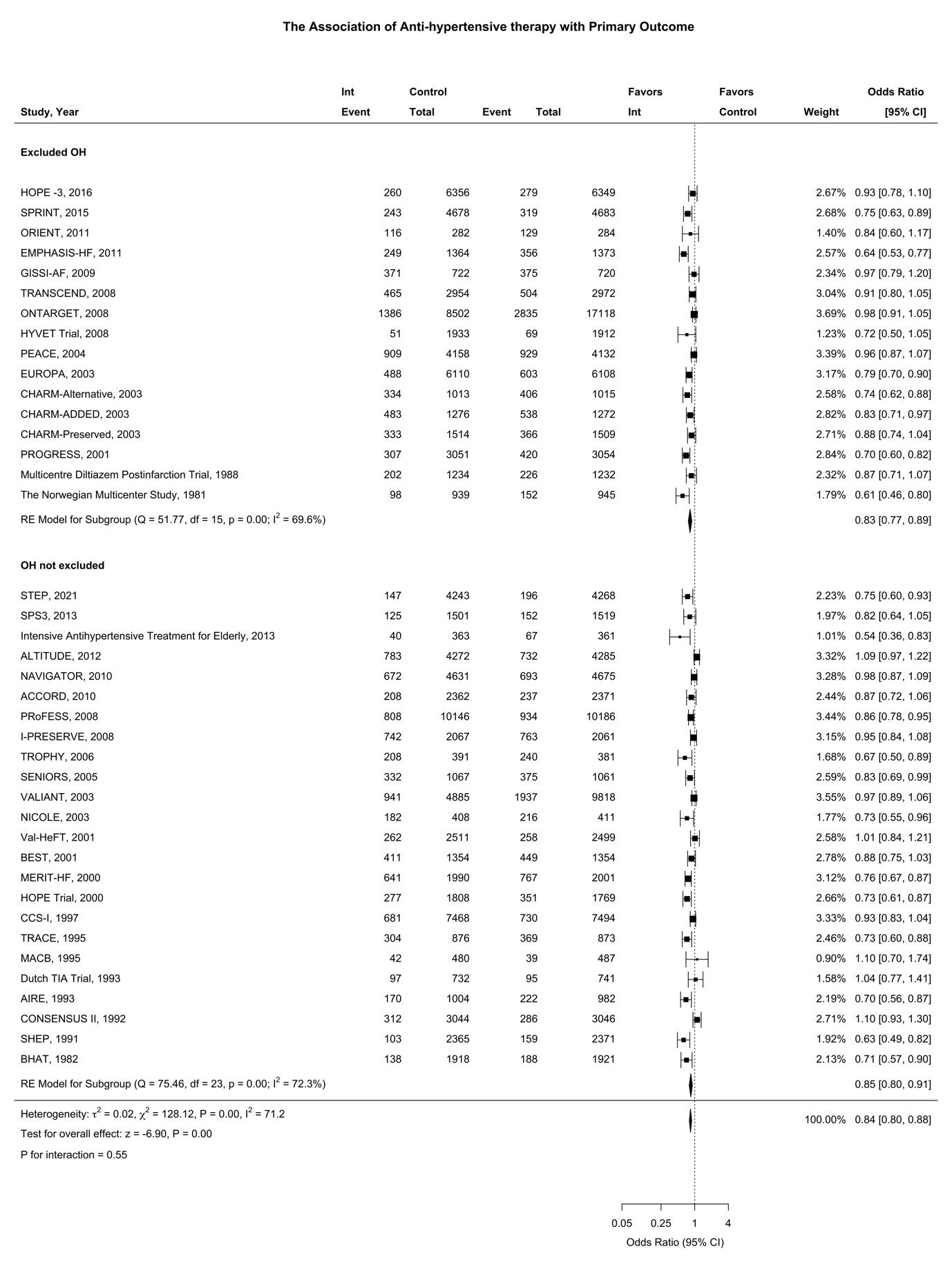


eFigure 7 – Forest plot demonstrating the association of anti-hypertensive therapy and primary outcome. The squares and bars represent the mean values and 95% confidence intervals of the effect sizes, while the area of the squares reflects the weight of the studies. The combined effects appear as diamonds and the vertical dashed line represents the line of no effect. Int-Intervention, CI-Confidence Interval, OH-Orthostatic hypotension

eFigure 8 – Risk of Bias


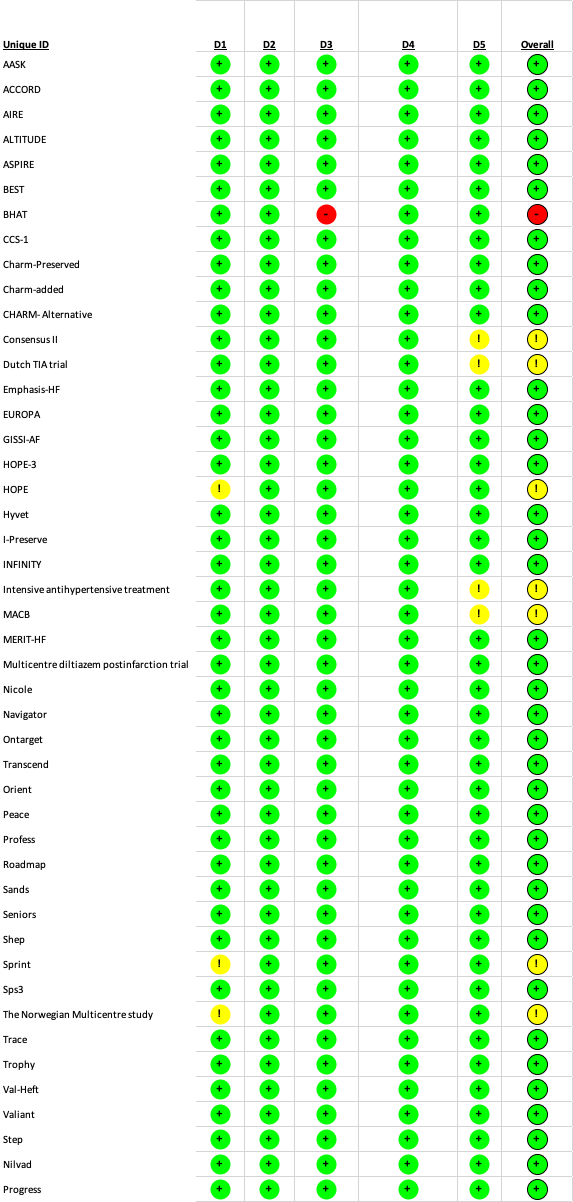


eFigure 9a – Funnel plot: OH not excluded trials


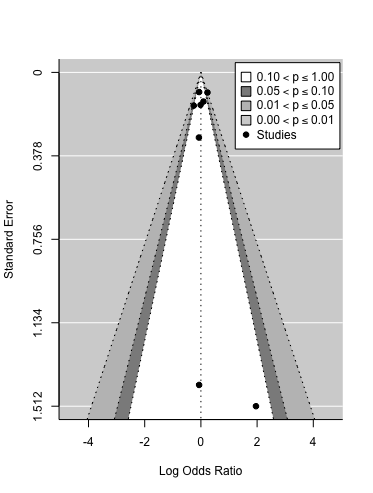


eFigure 9a – Contour enhanced funnel plot for trials which did not exclude OH, falls outcome. Different levels of statistical significance for studies are indicated by the shaded regions, detailed within the figure. The grey vertical line represents the summary estimate.

eFigure 9b – Funnel plot: OH excluded trials


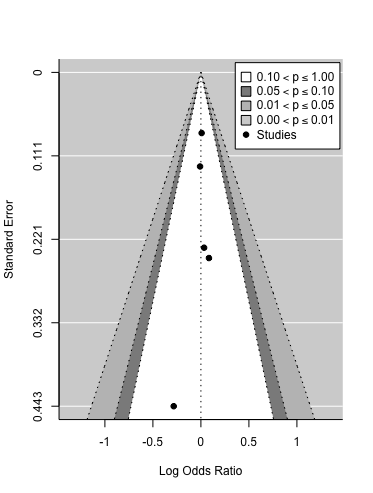


eFigure 9b – Contour enhanced funnel plot for trials which excluded OH, falls outcome. Different levels of statistical significance for studies are indicated by the shaded regions, detailed within the figure. The grey vertical line represents the summary estimate.

Tables

eTable 1 – Definition of trial primary outcomes and exclusion criteria applied where applicable

| **Trial name** | **Year** | **Primary outcome** | **OH exclusion criteria** |
| --- | --- | --- | --- |
| NILVAD | 2018 | Progression on the Alzheimer’s Disease Assessment Scale Cognitive Subscale-12 | Symptomatic OH in the last year |
| HOPE -3 | 2016 | Composite of death from cardiovascular causes, nonfatal myocardial infarction, or nonfatal stroke | Symptomatic hypotension (screening and run-in) |
| SPRINT | 2015 | First occurrence of myocardial infarction, acute coronary syndrome, stroke, heart failure, or death from cardiovascular causes. | Standing SBP <110 mm Hg at 1 minute |
| EMPHASIS-HF | 2011 | Death from cardiovascular causes or hospitalization for heart failure | Symptomatic hypotension (screening) |
| ORIENT | 2011 | Doubling of serum creatinine, ends-stage renal disease and death in type 2 diabetic patients with overt nephropathy | Severe orthostatic hypotension |
| GISSI-AF | 2009 | The time to the first recurrence of atrial fibrillation and the proportion of patients who had more than one episode of atrial fibrillation over the 1-year follow-up period | Standing systolic blood pressure < 110 mm Hg |
| SANDS | 2009 | Composite outcome of change in carotid intimal- medial thickness and cardiovascular events. | Drop >20 mm Hg on standing |
| HYVET Trial | 2008 | All stroke | Standing BP <140 |
| ONTARGET | 2008 | Death from cardiovascular causes, myocardial infarction, stroke, or hospitalization for heart failure. | Symptomatic hypotension (run-in) |
| TRANSCEND | 2008 | Composite of cardiovascular death, myocardial infarction, stroke, or hospitalisation for heart failure. | Symptomatic hypotension (run-in) |
| PEACE | 2004 | Death from CV causes, nonfatal MI, CABG, PCI | Symptomatic hypotension (run-in) |
| CHARM-Preserved | 2003 | Cardiovascular death or admission to hospital for heart failure | Symptomatic hypotension (screening) |
| CHARM-ADDED | 2003 | Cardiovascular death or unplanned admission to hospital for the management of worsening CHF | Symptomatic hypotension (screening) |
| CHARM-Alternative | 2003 | Composite of cardiovascular death or hospital admission for CHF | Symptomatic hypotension (screening) |
| EUROPA | 2003 | Cardiovascular death, myocardial infarction, or cardiac arrest | Symptomatic hypotension (run-in) |
| PROGRESS | 2001 | All stroke | Symptomatic hypotension (run-in) |
| Multicentre Diltiazem Postinfarction Trial | 1988 | Total mortality, death from cardiac cause, non-fatal MI | Symptomatic hypotension |
| The Norwegian Multicenter Study | 1981 | Mortality | Standing BP <100 |
| STEP | 2021 | The primary outcome was a composite of stroke, acute coronary syndrome (acute myocardial infarction and hospitalization for unstable angina), acute decompensated heart failure, coronary revascularization, atrial fibrillation, or death from cardiovascular causes | NA |
| INFINITY | 2019 | Change in gait speed and accrual of white matter hyperintensity volume after 3 years | NA |
| Intensive Antihypertensive Treatment for Elderly | 2013 | combined incidence of fatal/nonfatal stroke, acute myocardial infarction, and other cardiovascular deaths (sudden death and heart failure death). | NA |
| SPS3 | 2013 | Recurrent stroke | NA |
| ALTITUDE | 2012 | Composite of the time to cardiovascular death or a first occurrence of cardiac arrest with resuscitation; nonfatal myocardial infarction; nonfatal stroke; unplanned hospitalization for heart failure; end-stage renal disease, death attributable to kidney failure, or the need for renal-replacement therapy with no dialysis or transplantation available or initiated; or doubling of the baseline serum creatinine level | NA |
| ASPIRE | 2011 | Change in left ventricle end systolic volume from baseline to 36 weeks | NA |
| ROADMAP Trial | 2011 | Time to onset of microalbuminuria | NA |
| ACCORD | 2010 | Composite of nonfatal MI, nonfatal stroke, or death from cardiovascular causes | NA |
| NAVIGATOR | 2010 | An extended cardiovascular outcome, a composite of death from cardiovascular causes, nonfatal myocardial infarction, nonfatal stroke, hospitalization for heart failure, arterial revascularization, or hospitalization for unstable angina. | NA |
| I-PRESERVE | 2008 | Death from any cause or hospitalisation for a cardiovascular cause | NA |
| PRoFESS | 2008 | Recurrent stroke | NA |
| TROPHY | 2006 | Development of clinical hypertension | NA |
| SENIORS | 2005 | All-cause mortality or cardiovascular hospital admission | NA |
| NICOLE | 2003 | Cardiovascular events, including death, stroke, acute myocardial infarction, repeat PTCA | NA |
| VALIANT | 2003 | Mortality | NA |
| AASK | 2002 | Mean GFR decline | NA |
| BEST | 2001 | All-cause mortality | NA |
| Val-HeFT | 2001 | Mortality | NA |
| HOPE Trial | 2000 | Myocardial infarction, stroke, or cardiovascular death | NA |
| MERIT-HF | 2000 | Mortality or all-cause hospitalization | NA |
| CCS-I | 1997 | Mortality | NA |
| MACB | 1995 | death, non-fatal myocardial infarction, unstable angina pectoris, need for coronary artery bypass grafting or percutaneous transluminal angioplasty | NA |
| TRACE | 1995 | Death from any cause | NA |
| AIRE | 1993 | All-cause mortality | NA |
| Dutch TIA Trial | 1993 | Combined occurrence of death from vascular causes, nonfatal stroke, or nonfatal myocardial infarction, whichever occurred first | NA |
| CONSENSUS II | 1992 | Mortality | NA |
| SHEP | 1991 | All stroke | NA |
| BHAT | 1982 | Mortality | NA |
